# Sentinel lymph node biopsy versus lymphadenectomy in early-stage cervical cancer: a meta-analysis of oncologic outcomes and surgical morbidity

**DOI:** 10.3389/fonc.2026.1765529

**Published:** 2026-03-04

**Authors:** Chao Xiao, Siyuan Zeng, Luying Li, Ruiqi Wang, Xue Xiao

**Affiliations:** 1 Department of Obstetrics and Gynecology, West China Second University Hospital, Sichuan University, Chengdu, China; 2 Department of Obstetrics and Gynecology, The First People’s Hospital of Zigong, Zigong, China; 3 Key Laboratory of Birth Defects and Related Diseases of Women and Children (Sichuan University), Ministry of Education, West China Second Hospital, Sichuan University, Chengdu, China; 4 Tianfu Jincheng Laboratory, Chengdu, China; 5 Laboratory of Stem Cell & Embryo Development, West China Second Hospital, Sichuan University, Chengdu, China

**Keywords:** cervical cancer, cervical carcinoma, lymphadenectomy, meta - analysis, sentinel lymph node

## Abstract

**Objective:**

This study aimed to evaluate the oncologic safety of sentinel lymph node biopsy (SLNB) compared with systematic lymph node dissection (LND) in patients with early-stage cervical cancer and to determine whether SLNB alone yields comparable survival outcomes.

**Data sources:**

Studies published up to October 2025 were systematically searched in PubMed, Embase, and Web of Science using relevant keywords, including “sentinel lymph node”, “cervical cancer”, “cervical carcinoma” and “lymphadenectomy.”

**Study eligibility criteria:**

Comparative cohort studies and single-arm studies involving patients with early-stage cervical cancer undergoing SLNB, with or without LND, and reporting survival outcomes— including cancer-specific survival (CSS), disease-specific survival (DSS), overall survival (OS), progression-free survival (PFS), and disease-free survival (DFS) —were included.

**Study appraisal and synthesis methods:**

The quality of the included studies was assessed using appropriate tools: the Cochrane Risk of Bias 2.0 (RoB 2) tool for randomized controlled trials, the Newcastle–Ottawa Scale (NOS) for observational studies, and the Methodological Index for Non-Randomized Studies (MINORS) for single-arm or non-randomized studies. All meta-analyses were performed using the meta package in R. Hazard ratios (HRs) and risk ratios (RRs) with 95% confidence intervals (CIs) were pooled using fixed- or random-effects models depending on heterogeneity. Sensitivity analyses were conducted via leave-one-out analysis.

**Results:**

The pooled analysis of six comparative studies revealed no significant difference in cancer-specific survival (HR = 0.93, 95% CI: 0.27–3.20), overall survival (HR = 0.92 (95% CI: 0.65–1.31), disease-free survival (HR = 0.99, 95%CI: 0.00–855.48), or progression-free survival (HR = 0.71, 95% CI: 0.29-1.05) between the SLNB and LND groups. SLNB was associated with a significantly lower risk of postoperative complications (RR = 0.70, P = 0.0406), and did not increase the recurrence rate (RR = 0.96, 95% CI: 0.36-2.53) compared with LND. Six single-arm studies reported 5-year OS and DFS rates of 97% and 94%, respectively, following SLNB alone. The pooled SLNB positivity rate across 13 studies was 8% (95% CI: 5%–12%). Sensitivity analysis confirmed the robustness of the CSS results.

**Conclusion:**

This study suggests that SLNB provides oncologic outcomes comparable to LND while reducing surgical morbidity in early-stage cervical cancer. The inclusion of CSS as a validated endpoint reinforces the cancer-specific safety of SLNB, with no significant compromise observed in either OS or PFS. While current evidence is promising, further large-scale prospective trials are needed to refine indications and standardize implementation of SLNB in routine clinical practice.

## Introduction

Cervical cancer remains a leading cause of cancer-related morbidity and mortality among women worldwide, especially in low- and middle-income countries (1, 2). While early-stage cervical cancer (FIGO IA2–IIA1) is potentially curable with radical hysterectomy and lymphadenectomy, the optimal extent of nodal assessment has been a topic of considerable clinical debate (3, 4). Historically, systematic pelvic lymph node dissection (LND) has served as the standard staging and therapeutic procedure, enabling detection of occult metastases and informing adjuvant treatment decisions. However, this approach is associated with significant short- and long-term complications, including lymphocyst formation, lymphedema, neurovascular injury, and prolonged recovery (5, 6).

Sentinel lymph node biopsy (SLNB) has emerged as a less invasive alternative, aiming to maintain diagnostic accuracy while minimizing surgical morbidity. The SLNB technique, initially established in breast cancer and melanoma, has gained traction in gynecologic oncology due to advancements in tracers (e.g., indocyanine green), imaging, and ultrastaging pathology (3, 7). Several prospective and retrospective studies have reported high sensitivity, negative predictive value, and bilateral detection rates with SLNB in early cervical cancer (5, 8–12). However, concerns remain regarding false-negative rates, especially in patients with large tumors, lymphovascular space invasion (LVSI), or non-standard SLNB drainage.

Recent randomized controlled trials, such as the PHENIX and SENTICOL studies, suggest that SLNB alone may offer non-inferior oncologic outcomes compared to full pelvic LND, while significantly reducing perioperative and long-term complications (5, 13–15). Nevertheless, clinical guidelines remain cautious, and consensus is lacking on whether SLNB alone can safely replace systematic LND across all patient subsets (14, 16, 17).

Given the growing body of evidence and emerging data from high-quality studies, a systematic review and meta-analysis is warranted to synthesize current findings. This study integrates data from randomized trials and prospective cohorts to compare SLNB and pelvic LND in early-stage cervical cancer, with a primary focus on oncologic safety—as measured by cancer-specific survival (CSS)—and a secondary focus on overall survival (OS), progression-free survival (PFS), disease-free survival (DFS), recurrence rate and postoperative morbidity. The results will help clarify the role of SLNB in standard surgical management and inform future clinical guidelines.

## Methods

### Search strategy and study selection

This meta-analysis was conducted in accordance with the PRISMA guidelines with the registration (18). A comprehensive literature search was performed in PubMed, Embase, and Web of Science from inception to October 2025. Search terms included combinations of “sentinel lymph node”, “SLNB”, “cervical cancer”, “cervical carcinoma” and “lymphadenectomy”. Eligible studies were comparative or single-arm designs reporting survival outcomes in early-stage cervical cancer patients who underwent SLNB, with or without LND. Duplicate records were removed, and titles, abstracts, and full texts were screened independently by two reviewers.

### Inclusion and exclusion criteria

Studies were included if they: (1) involved patients with early-stage cervical cancer (FIGO 2009 stage IA1–IIA1); (2) reported at least one of the following outcomes: CSS, DSS, OS, PFS, and DFS, or SLNB positivity rate; and (3) provided sufficient data to calculate hazard ratios (HRs) or risk ratios (RRs) with 95% confidence intervals (CIs). Reviews, case reports, and studies with overlapping populations were excluded.

### Data extraction and quality assessment

Two independent reviewers extracted data on study characteristics, patient demographics, surgical techniques, and survival outcomes. For consistency across studies, DSS data were treated as equivalent to CSS and were pooled under the CSS category during meta-analysis. Discrepancies were resolved by consensus or a third reviewer. For comparative studies, HRs and RRs with 95% CIs were extracted directly or estimated using Tierney’s method when not explicitly reported. For single-arm studies, proportions and corresponding 95% CIs were calculated. The Cochrane Risk of Bias 2.0 tool was used for randomized controlled trials (RCTs) (19), the Newcastle-Ottawa Scale (NOS) for observational studies (20), and the Methodological Index for Non-Randomized Studies (MINORS) for single-arm or non-randomized studies (21). Risk of bias assessment was independently conducted by two reviewers.

### Statistical analysis

All statistical analyses were performed using R (version 4.5.1) within RStudio, utilizing the ‘meta’ and ‘metafor’ packages for pooled effect size estimation, forest plot generation, and sensitivity analyses. Pooled HRs and RRs were calculated using fixed-effects models when heterogeneity was low (I² < 50%) and random-effects models otherwise. Heterogeneity was assessed using the I² statistic and Chi² test. Sensitivity analyses were conducted via leave-one-out meta-analysis to test the robustness of the pooled results. Publication bias was not formally assessed due to the limited number of studies for each outcome.

## Results

### Study selection

**Figure 1:**
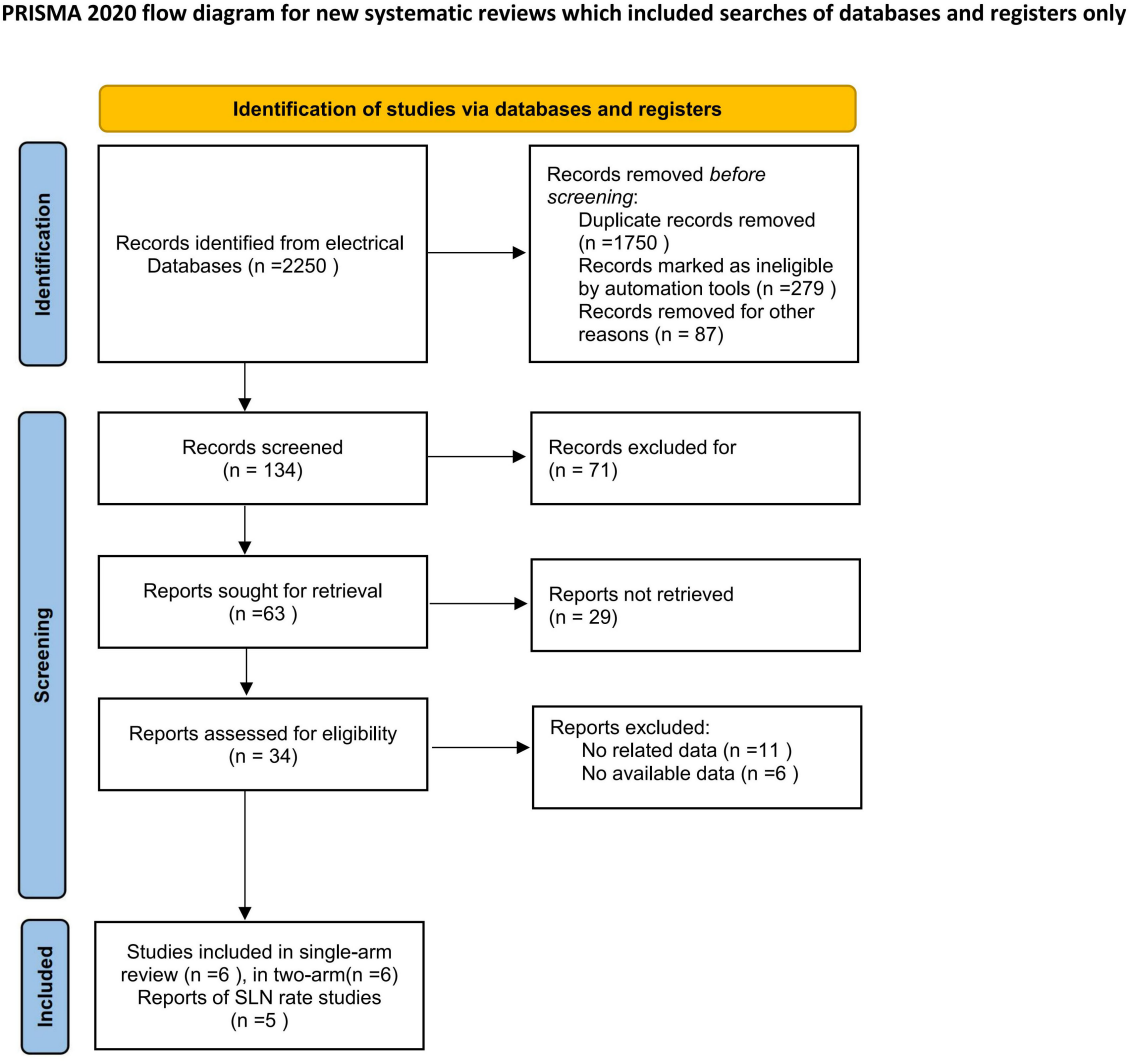
Flow plot of the literature selection process.

Of 2,250 records identified from databases, 1,750 duplicates and 366 ineligible or irrelevant records were removed. After screening 134 records, 71 were excluded. Of the 63 reports sought for retrieval, 29 were unavailable. Following full-text assessment of 34 articles, 17 were excluded due to unrelated or unavailable data. A total of 17 studies were included: 6 for single-arm meta-analysis, 6 for two-arm meta-analysis, and another 5 reporting sentinel lymph node rates. The literature retrieval process is illustrated in **Figure 1**.

### Study characteristics

The meta-analysis included a total of 3,855 patients, comprising 1,156 patients from six single-arm studies and 2,699 patients from five comparative studies. Among the comparative studies, 1,239 patients underwent sentinel lymph node biopsy (SLNB) and 1,460 patients underwent lymphadenectomy (LND), with or without concurrent SLNB (**Table 1**).

**Table 1:** Characteristics of studies included in this meta-analysis.

Study, year	Study design	Country	Duration	Sample size (SLNB/LND)	Age (SLNB/LND)	Median follow-up (months)	Stage(year)	Summary statistics
Tu H et al., 2025 (5)	RCT	China	December 2015-December 2023	420/418	48/49	62.8(8.9-110.1)	IA1(LVSI), IA2, IB1, IIA1	3-year DFS(SLNB 96.9% vs LND 94.6%)HR=0.61 (95% CI: 0.33–1.14);CSS (SLNB 96.9% vs LND 97.8%)HR = 0.37 (95% CI: 0.15–0.95);Recurrence(SLNB 16/420 vs LND 26/418);All complications (244/420 vs298/418)
Martin et al., 2025 (11)	Retrospective	Spain	SLN + PLD group(2001–2011) SLN-only group(2012–2022)	112/98	43.3/45.4	80(3–275)	IA1 – IIA1	3-year PFS:97.2% vs 93.7%HR=0.66(95%CI0.22 -2.04);3-year OS:99.0% vs 98.9%HR=0.28(95%CI0.03 -2.53);Recurrence(SLN+PLD 8/98 vs SLN 5/112;)Positive SLN Rate:23/210 (11%)
Friedman et al., 2025 (22)	Retrospective cohort	United States	2004–2021	365/391	42/43	SLNB 28.8/LND50.4	AJCC T1 (T1a and T1b)	5-year CSS (SLNB 96.5% vs LND 95.7%)HR = 1.25 (95% CI: 0.45–3.47);5-year OS:95.3% vs 94.6%HR=0.96(95%CI0.41 -2.25)
Balaya V et al., 2022 (15)	Prospective	France	January 2005 – July 2012	87/172	43.9/43.3	53(5–85)/46(4–127)	IA1(LVSI), IA2, IB1, IB2, IIA1	7-year DFS(SLNB 85.1% vs LND 80.4%)HR = 1.78 (95% CI: 0.71–4.46); DSS(SLNB 90.8% vs LND 97.2%)HR = 3.02 (95% CI: 0.69–13.18); Recurrence(SLNB 10/87 vs LND 11/172)
Matsuo et al., 2022 (12)	Retrospective	United States	2003-2018	150/280	45/44	SLNB23/LND84	IA(119)IB(305)	5-year OS:94.8% vs 94.2%, HR: 0.95, 95% CI: 0.64-1.41, p=0.799);CSS: HR 0.90,95% CI: 0.49–1.64,P = 0.720;
Mathevet P et al., 2021 (13)	RCT	France	March 2009-June 2012	105/101	44.2/44.6	36	IA1 (with LVSI), IA2, IB1 (majority), IIA1	3-year PFS(SLNB 92% vs LND 94%, HR = 0.76 (95% CI: 0.25–2.34)Log-rank: p=0.48);Recurrence(SNB 8/105 vs PLND 5/101);All complications (33/105 vs 56/101)
Gortzak-Uzan et al., 2010 (10)	Retrospective cohort	Canada	2000–2008	81	38.2	13	IA:32 IB:49	DFS: 91%;Positive SLN rate:14( 17.3%)
Lennox et al., 2017 (23)	Prospective	Canada	NA	110	35	32	IA(32) and IB(45)	DFS:93%;Positive SLN rate:0
Balaya et al., 2022 (15)	Prospective	France	2005-2016	87	42	53	IA(9)IB(74)IIA1(3)IIB(1)	DFS: 85.1%;OS: 90.8%;Positive SLN rate:0
Devaja et al., 2022 (9)	cohort	United Kingdom	January 2009-January 2019	103	36	53	IA1 (with LVSI), IA2, IB1 (tumor <2 cm)	DFS:93%;OS:99.1%;Positive SLN rate:7 (6.7%)
Yahata et al., 2022 (24)	cohort	Japan	2009-2017	181	34	83.5	IA(24)IB(154)IIA1(3)	DFS:98.9%;OS:99.4%;Positive SLN rate:8(4.4%)
Cibula et al., 2025 (8)	cohort	Multinational	July 2016-November 2020	594	43.8	47	IA1 with LVSI+, IA2, IB1, IB2 (Majority: IB1 53%, IB2 31%)	5-year DFS:89.9%;5-year OS:93.4%;Positive SLN rate: 9%
Lührs et al., 2021 (6)	cohort	Sweden	2018-2021	145	43.6	NA	IA1 (with LVSI), IA2, IB1 (majority), few IB2 and IIA included	Positive SLN rate: 19/145 (13.1%)
Ya et al., 2021 (35)	Prospective	China	May 2017 – December 2019	325(SLNB+LM)	46	NA	Ia2–IIa2	Positive SLN rate:44(12.3%)
Tu et al., 2020 (26)	prospective	China	May 2014 - June 2016	75(SLNB+LM)	46	53	IA2, IB1, IB2, IIA1, IIA2, IIB	Positive SLN rate:11(14.7%)
Mayoral et al., 2017 (36)	prospective	Spain	June 2011 - January 2013	17(SLNB+LM)	53.3	26	IA1 (1 patient), IB1 <2 cm (16 patients)	Positive SLN rate:1(5.8%)
Bats et al., 2007 (25)	Prospective	France	January 2003-March 2006	25(SLNB+LM)	51.2 ± 16.4	NA	IA2;IB1 <2 cm:	Positive SLN rate:2(8%)

Studies varied in geographic origin, sample size (range: 81–838), and follow-up duration (median range: 13–83.5 months). Most studies employed dual tracers for SLNB, and ultrastaging pathology was applied in five studies. All studies enrolled patients with early-stage cervical cancer (FIGO IA2–IIA1).

### Quality assessment of included studies

The two randomized controlled trials assessed using the RoB 2.0 tool were judged to have an overall low risk of bias. Four observational studies evaluated with the Newcastle–Ottawa Scale achieved high-quality scores. Non-randomized and single-arm six studies assessed with the MINORS tool demonstrated fair to moderately high quality, indicating generally reliable but design-limited evidence. Overall, the methodological quality of the included studies was acceptable (**Table 2**).

**Table 2:** Quality assessment of included studies.

Quality assessment table							
RoB 2.0 – Cochrane risk of bias tool for randomized trial							
Study	Study design	Randomization (low/high risk)	Intervention bias (low/high risk)	Missing data bias (low/high risk)	Outcome measurement bias (low/high risk)	Reporting bias (low/high risk)	Overall risk of nias
Tu H et al., 2025 (5)	RCT	Low	Low	Low	Low	Low	Low
Mathevet P et al., 2021 (13)	RCT	Low	Low	Low	Low	Low	Low
Newcastle-Ottawa Scale (NOS)							
Study	Selection	Comparability	Outcome	Total Score	Quality		
Friedman et al., 2025 (22)	4	2	2	8	High		
Matsuo et al., 2022 (12)	4	2	3	9	High		
Martin et al., 2025 (11)	4	2	3	9	High		
Balaya V et al., 2022 (15)	4	2	3	9	High		
Methodological index for non-randomized studies							
Author	Prospective	MINORS Score	Quality				
Devaja et al., 2022 (9)	Yes	11	Moderate				
Lührs et al., 2021 (6)	Yes	10	Moderate				
Gortzak-Uzan et al., 2010 (10)	Yes	8	Fair				
Lennox et al., 2017 (23)	Yes	9	Moderate				
Balaya et al., 2022 (15)	Partial	13	Moderately High				
Yahata et al., 2022 (24)	No	11	Moderate				

### Oncologic outcomes in two-arm studies

**Figure 2:**
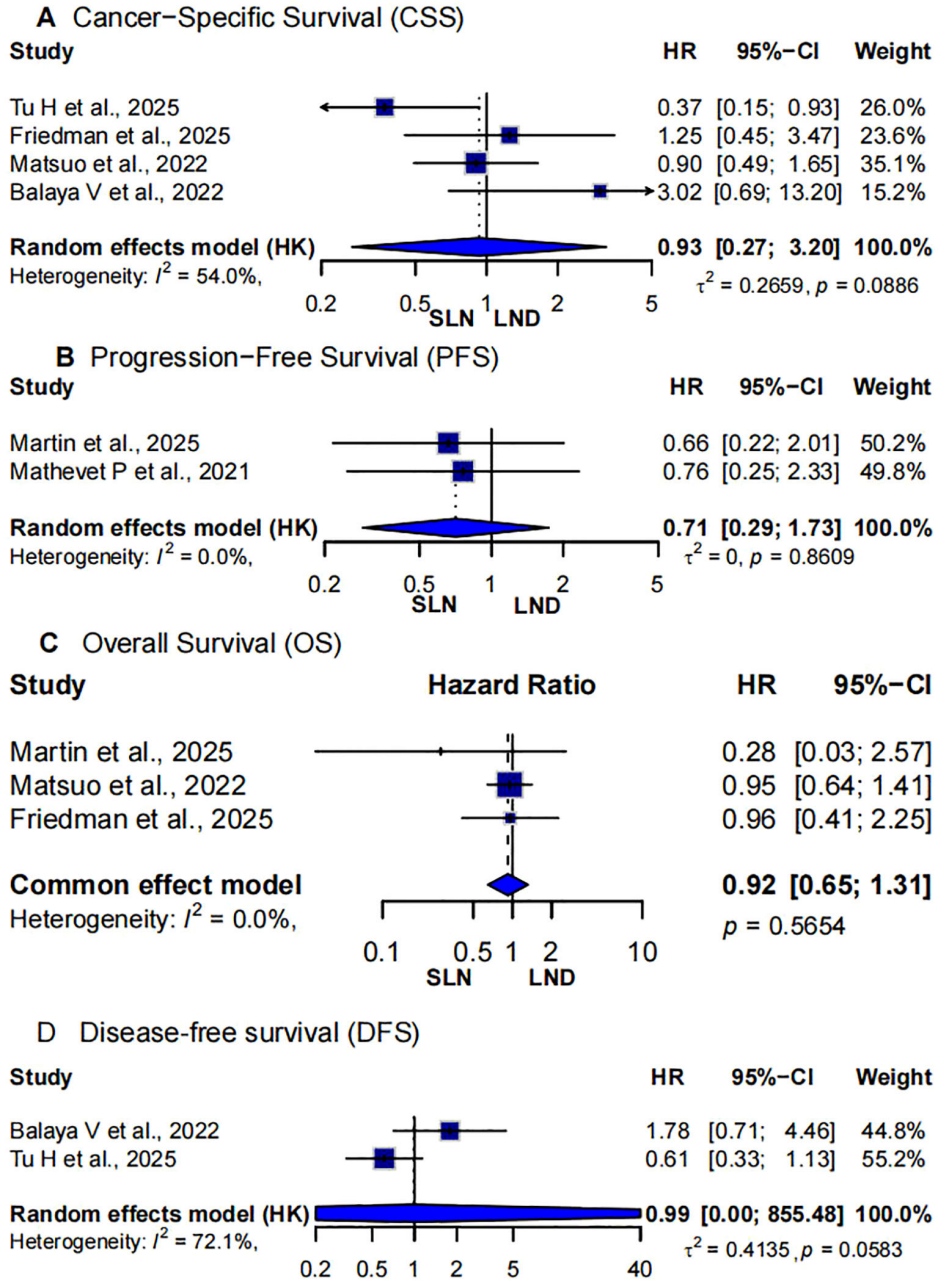
Forest plot of oncologic outcomes in two-arm studies. **(A)** cancer-specific survival (CSS); **(B)** progression-free survival (PFS); **(C)** OS, overall survival; SLNB, Sentinel Lymph Node Biopsy; LND, lymph node dissection.

Four studies compared cancer-specific survival between SLNB and LND in early-stage cervical cancer patients (5, 12, 15, 22). Due to moderate heterogeneity (I² = 54.0%, p=0.0886), a random-effects model was used. The pooled hazard ratio was 0.93 (95% CI: 0.27-3.20), indicating no statistically significant difference in CSS between SLNB and LND, although the point estimate leaned slightly in favor of SLNB (**Figure 2A**).

Three studies reported hazard ratios for progression-free survival (PFS) (11, 13). All individual studies showed non-significant results but tended to favor SLNB. The pooled estimate from a fixed-effect model (due to zero heterogeneity, I² = 0.0%) yielded a hazard ratio of 0.71 (95% CI: 0.29-1.05, p=0.86). This suggests no statistically significant difference in PFS between SLNB and LND (**Figure 2B**).

A meta-analysis of three studies evaluating overall survival (OS) demonstrated low heterogeneity (I² =0.0%), justifying the use of a fixed-effect model (11, 12, 22). The pooled hazard ratio was 0.92 (95%CI: 0.65-1.31, p=0.5654), indicating no significant survival difference between SLNB and LND (**Figure 2C**).

Two studies assessed disease-free survival (DFS) (5, 15). The combined analysis using a random-effects model due to high heterogeneity (I² = 72.1%, p = 0.0583) yielded a hazard ratio of 0.99 (95% CI: 0.00–855.48). The extremely wide confidence interval reflects instability due to the limited number of studies and significant between-study variability. These results suggest substantial uncertainty in the true comparative effectiveness of SLNB versus LND for DFS (**Figure 2D**).

### Surgical morbidity and recurrence

**Figure 3:**
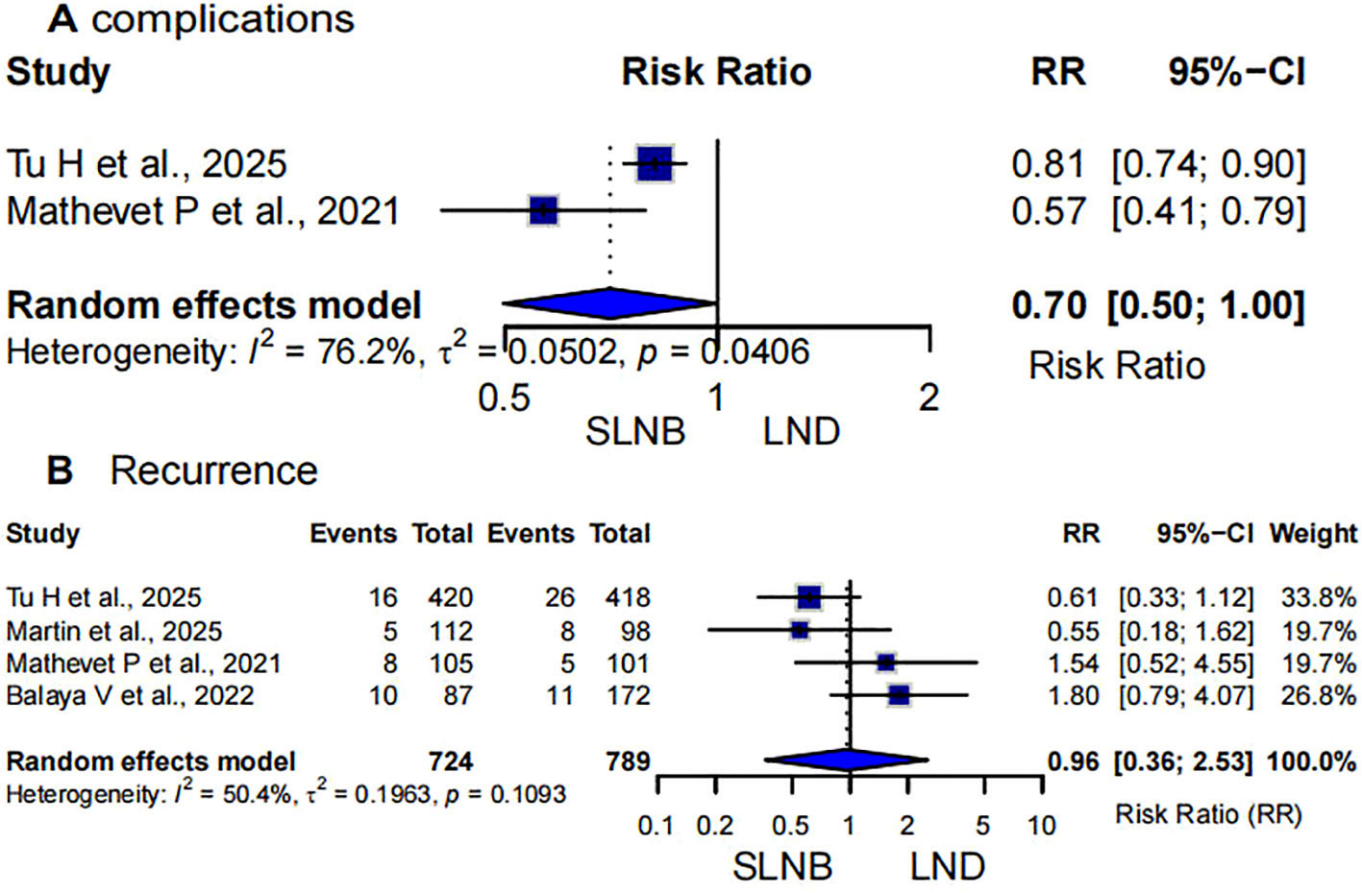
Forest plot of **(A)** complications and **(B)** recurrence rate. SLNB Sentinel Lymph Node Biopsy; LND, lymph node dissection.

Two studies evaluated the risk of postoperative overall complications following SLNB versus LND (5, 13). The meta-analysis showed that patients undergoing SLNB had a significantly lower risk of postoperative complications compared to those receiving systematic lymphadenectomy, with a pooled risk ratio (RR) of 0.70 (95% CI: 0.50–1.00, p = 0.0406). The heterogeneity among studies was substantial (I² = 76.2%), and a random-effects model was applied. These findings suggest that SLNB may significantly reduce postoperative morbidity in early-stage cervical cancer, although variability between studies warrants cautious interpretation (**Figure 3A**).

Four studies compared recurrence rates between SLNB and LND groups (5, 11, 13, 15). The pooled analysis using a random-effects model demonstrated no statistically significant difference in recurrence risk between the two surgical approaches (RR = 0.96, 95% CI: 0.36-2.53, p=0.1093). Moderate heterogeneity was observed (I² =50.4%), suggesting variability in effect estimates across studies. Although the pooled point estimate slightly favored SLNB, the wide confidence intervals indicate considerable uncertainty. Overall, these results suggest that SLNB may provide a recurrence risk comparable to LND in early-stage cervical cancer patients (**Figure 3B**).

### Oncologic outcomes in single-arm studies

**Figure 4:**
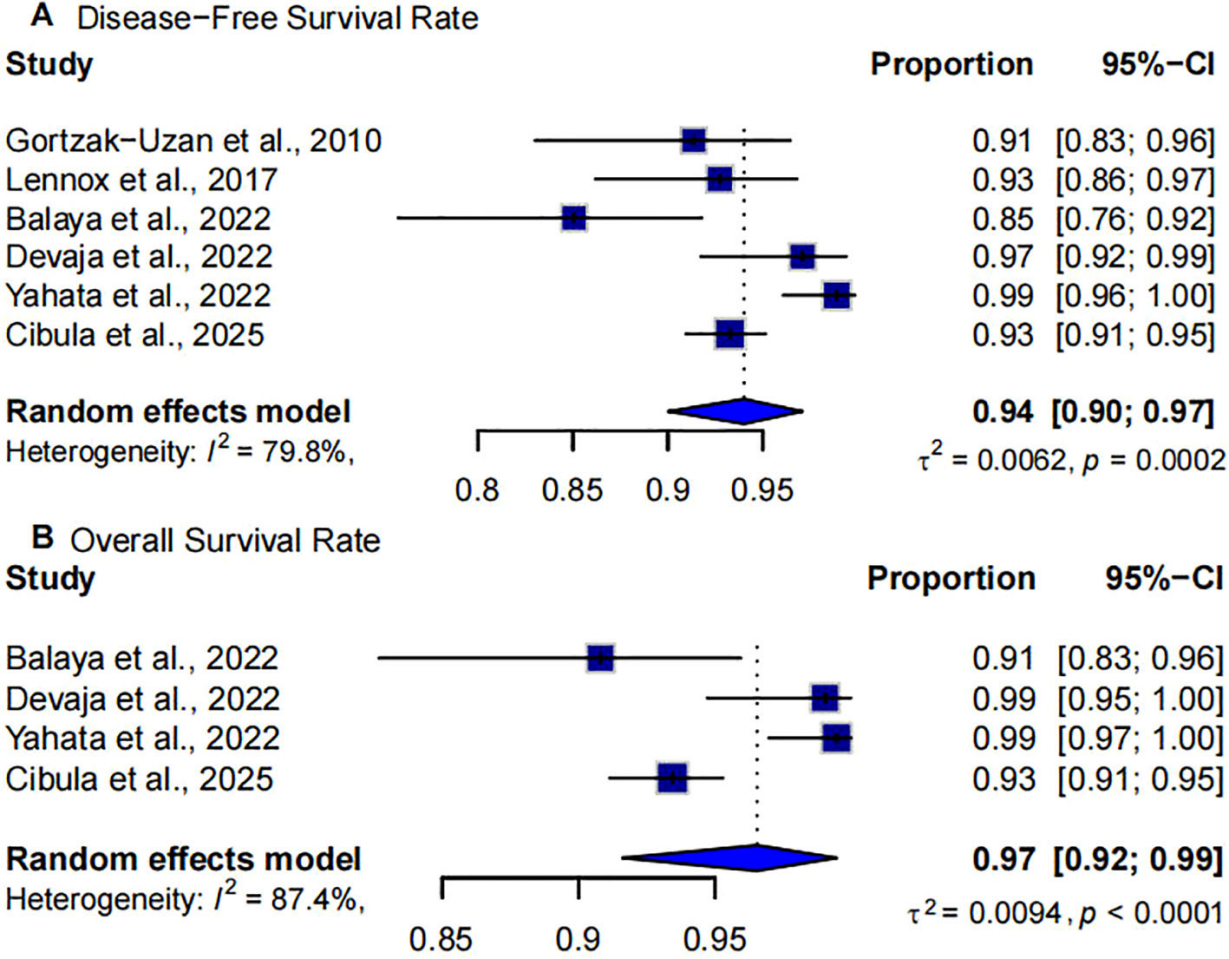
Forest plot of oncologic outcomes in single-arm studies. **(A)** disease-free survival (DFS); **(B)** overall survival (OS).

Six single-arm studies reported disease-free survival (DFS) rates following SLNB in early-stage cervical cancer (8–10, 15, 23, 24). The pooled 5-year DFS rate was 94% (95% CI: 90%–97%), reflecting favorable long-term disease control with SLNB alone. Despite the strong overall estimate, substantial heterogeneity was observed (I²=79.8%, p=0.0002), likely due to differences in study design, population risk profiles, and follow-up durations (**Figure 4A**).

Four single-arm studies evaluated OS following SLNB in patients with early-stage cervical cancer (8, 9, 15, 24). The pooled 5-year OS rate was 97% (95% CI: 92%-99%), suggesting favorable long-term survival outcomes with SLNB alone. However, heterogeneity across studies was high (I²=87.4%, p < 0.0001), indicating variability in patient populations or study protocols (**Figure 4B**).

### Sensitivity analysis of cancer-specific survival

**Figure 5:**
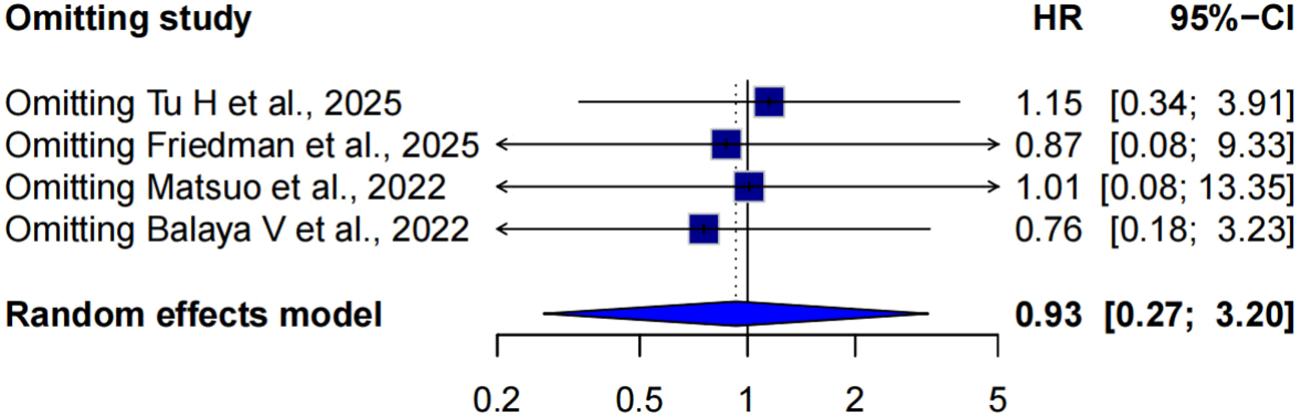
Forest plot of a leave-one-out sensitivity analysis across three studies using a random-effects model.

A leave-one-out sensitivity analysis was conducted to assess the robustness of the pooled hazard ratio (HR) for cancer-specific survival (CSS) under a random-effects model. Sequential omission of each study yielded pooled HRs ranging from 0.76 to 1.15, with all 95% confidence intervals encompassing the null value (HR = 1.0), indicating no significant change in the overall conclusion. Notably, omission of Tu H et al. (5) resulted in the highest pooled HR of 1.15 (95% CI: 0.34–3.91), suggesting that this study had a substantial influence on the observed benefit of SLNB. In contrast, exclusion of Balaya et al. (15) yielded the lowest pooled HR of 0.76 (95% CI: 0.18–3.23), slightly reinforcing the advantage of SLNB. Overall, these findings indicate that the observed effect (pooled HR = 0.93, 95% CI: 0.27-3.20) is not driven by any single study and is therefore robust (**Figure 5**).

### Sentinel lymph node positivity rate

**Figure 6:**
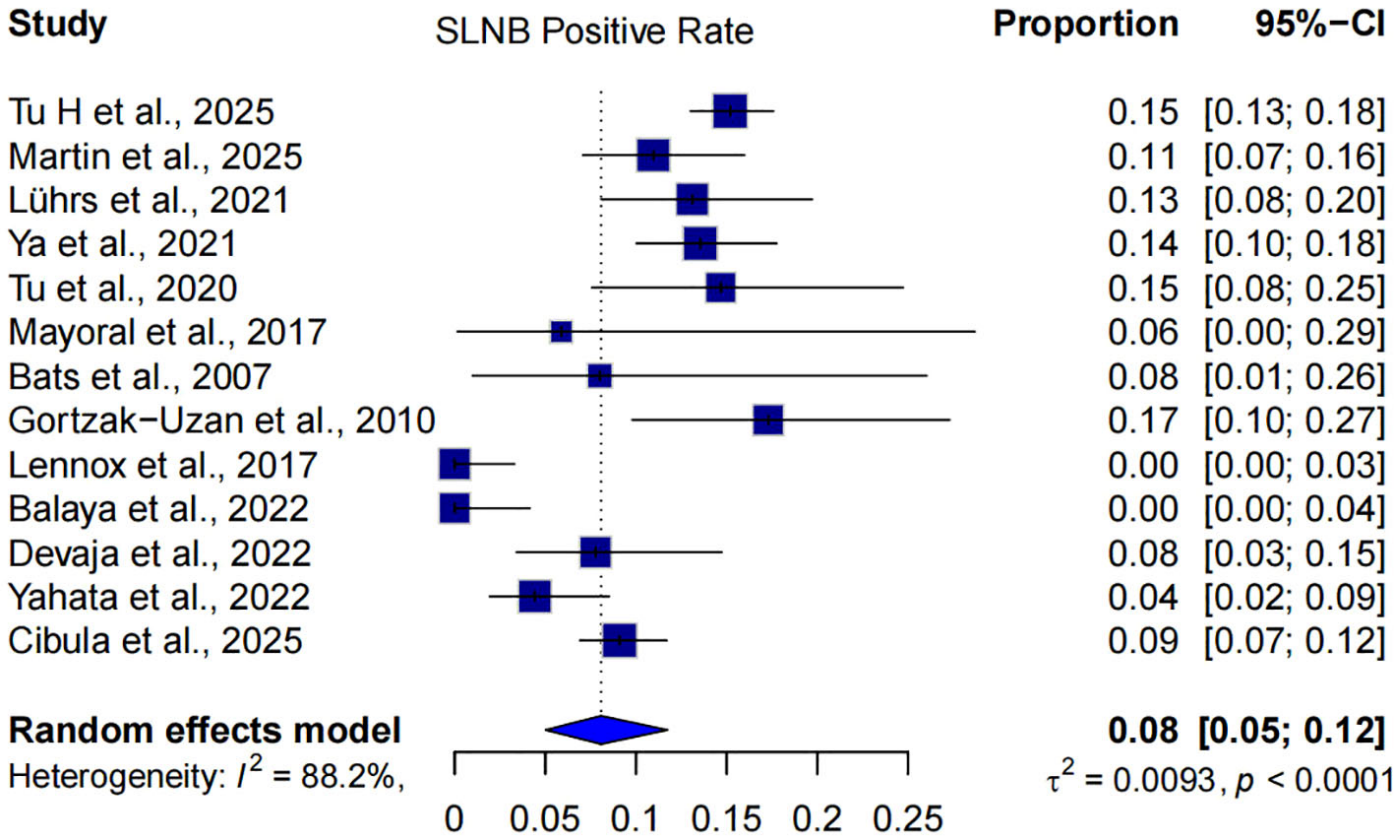
Forest plot of SLNB positivity rate of 13 studies using a random-effects model.

A total of 13 studies (including both single-arm and comparative designs) reported the sentinel lymph node (SLN) positivity rate in patients with early-stage cervical cancer undergoing SLNB (5, 6, 8–13, 15, 23–26). The pooled SLNB positivity rate was 8% (95% CI: 5%-12%), based on a random-effects model. Substantial heterogeneity was observed across studies (I²=88.2%, p < 0.0001), likely reflecting differences in patient selection, tumor characteristics, and SLNB protocols (**Figure 6**).

## Discussion

### Summary of main findings

This meta-analysis comprehensively evaluated the oncologic safety of SLNB in early-stage cervical cancer, integrating evidence from both comparative and single-arm studies. Across six controlled studies comparing SLNB with systematic LND, no significant differences were found in cancer-specific survival (CSS, HR = 0.93), overall survival (OS, HR = 0.92), progression-free survival(PFS, HR = 0.71), recurrence rate(RR,RR = 0.96), or disease-free survival (DFS, HR = 0.99). SLNB was, however, associated with a significantly lower risk of postoperative complications (RR = 0.70, p=0.0406), emphasizing its benefit in reducing surgical morbidity without compromising oncologic outcomes.

Importantly, this study places CSS at the forefront—a rarely reported yet clinically meaningful endpoint that excludes non-cancer mortality. By pooling CSS data from four high-quality studies, we obtained a more precise measure of oncologic safety, confirming that SLNB does not increase cervical cancer-specific mortality. This refined metric is particularly valuable in younger patients where competing mortality risks can obscure oncologic efficacy in OS analysis.

Sensitivity analysis confirmed the robustness of the CSS finding. Leave-one-out analysis showed that removal of any single study did not substantially alter the pooled HR (range: 0.27-3.20), and all confidence intervals remained non-significant. The slight shift observed after excluding Tu H et al. (5)(HR = 1.15) suggests moderate influence, but the overall conclusion remained unchanged.

To further strengthen the evidence base, we incorporated data from six single-arm studies evaluating SLNB alone. These showed pooled 5-year OS and DFS rates of 97% and 94%, respectively, confirming excellent long-term outcomes in patients managed without additional lymphadenectomy. These findings reinforce the premise that, in appropriately selected patients, SLNB alone provides durable disease control.

Additionally, analysis of 13 studies reporting SLNB positivity rates revealed a pooled detection rate of 8% (95% CI: 5%-12%), indicating a relatively low burden of nodal metastasis in this population. This further supports the feasibility of a limited-node strategy, as the majority of patients are unlikely to benefit from full pelvic LND.

In summary, our findings provide robust evidence that SLNB is oncologically safe and clinically advantageous, particularly when cancer-specific endpoints are considered. The combination of favorable survival outcomes, reduced surgical morbidity, and low nodal involvement suggests that SLNB may serve as an effective standalone staging approach in selected patients with early-stage cervical cancer.

### Comparison with previous literature

The primary outcome, CSS, did not differ significantly between the groups. Tu et al. showed a significant reduction in CSS favoring SLNB (HR: 0.37, 95% CI: 0.15–0.93) (5), whereas Friedman et al., Matsuo et al. and Balaya et al. reported non-significant associations (12, 15, 22). While previous large-scale meta-analyses have consistently established the equivalence of SLNB and systematic LND in OS and recurrence or PFS (27–29), direct pooled estimates for CSS have been notably absent. Our finding of non-inferior CSS (P = 0.088) is a pivotal contribution to address this gap directly, offering the most precise quantitative evidence to date that the SLNB strategy does not lead to an increase in cervical cancer-related mortality. This refined metric is especially critical for this patient population, as it isolates the oncologic efficacy of the procedure from competing risks of death, which can often obscure the true treatment effect in OS analyses of younger cohorts (30).

The excellent long-term survival outcomes observed in our study are strongly corroborated by high-quality prospective and comparative studies. The PHENIX trial demonstrated equivalent 3-year DFS between the LNB groups (94.9% vs 92.0%) (5). Similarly, the SENTICOL study, no significant difference was observed between the LNB and LND groups, with HR = 1.78 (95% CI: 0.71-4.46) for DFS and HR = 3.02 (95% CI: 0.69-13.18) for CSS (15). Another analysis reported both 5-year DFS and OS exceeding 90% after SLNB alone, with no significant difference from survival after full LND (29) (31). DFS estimates across studies ranged from 85% to 99%. While Balaya et al. (15) reported the lowest rate (85%, 95% CI: 76%–92%), Yahata et al. (24) and Devaja et al. (9) documented near-complete disease-free survival at 99% and 97%, respectively. Individual study estimates OS ranged from 91% to 99%, with Devaja et al. (9) and Yahata et al. (24) both reporting near-perfect survival rates (99%), while Balaya et al. (15) reported a lower estimate of 91% (95% CI: 83%-96%). Our pooled 5-year OS and DFS rates of 97% and 94% from single-arm studies further solidify these findings, confirming that durable disease control can be reliably achieved when SLNB alone is used to guide subsequent management in node-negative patients.

Furthermore, the significant reduction in postoperative complications (RR = 0.70) associated with SLNB that we identified reinforces a key clinical advantage of this approach. Previous meta-analysis detailed a marked decrease in intraoperative bleeding, lymphocyst formation, and lower-limb lymphedema, directly translating to a superior patient-reported quality of life (32). This reduction in surgical morbidity, without any compromise in oncologic outcomes, represents a major step forward in the surgical management of early-stage cervical cancer.

Several studies, including those by Lennox et al. (23), Balaya et al. (15), and Devaja et al. (9), reported extremely low positivity rates (0-4%), whereas others, such as Gortzak-Uzan et al. (10) and Tu et al. (5), reported rates exceeding 13%. Despite this variability, the overall pooled estimate reflects a relatively low rate of SLN metastasis in early-stage disease, reinforcing the clinical value of SLNB as a low-burden staging modality. The diagnostic performance of SLNB also remains robust in early-stage cervical cancer. A large multicenter trial reported an overall detection rate of 96.3% and a bilateral detection rate of 82.0% (33). Meta-analytic evidence further estimates the pooled side-specific sensitivity of SLNB to be approximately 88% (32, 34). Our analysis builds upon these results by incorporating both comparative and single-arm evidence and by integrating more recent data through 2025. This updated synthesis reinforces SLNB’s clinical utility as a precise and less invasive staging approach in early cervical cancer.

### Clinical implications

The findings of this meta-analysis have direct implications for surgical decision-making in early-stage cervical cancer. The comparable survival outcomes observed between SLNB and systematic LND—including CSS, DFS, OS, and PFS—demonstrate the oncologic safety of SLNB as a less invasive surgical strategy. Importantly, the significantly lower postoperative complication rate associated with SLNB highlights its potential to reduce treatment-related morbidity without compromising long-term survival.

These results are particularly relevant for patients with low-risk clinical profiles, in whom the probability of lymph node metastasis is low and the potential harm of over treatment is high. By limiting surgical extent, SLNB offers a alternative for selected women, aligning with the principles of precision medicine and patient-centered care. At the same time, performing less extensive surgery reduces physical strain on the surgeon.

Moreover, the inclusion of CSS as a primary endpoint strengthens the evidence base by isolating cancer-related mortality, making the oncologic equivalence of SLNB even more clinically credible. Given the accumulating data from both comparative and single-arm studies, SLNB may be reasonably considered as a standalone surgical approach in appropriately selected patients. These findings may inform future guidelines and promote broader adoption of SLNB in clinical practice, provided that institutional expertise and SLN mapping protocols are in place.

### Limitations and future directions

This meta-analysis has several limitations that warrant consideration. First, although multiple comparative studies were included, the total number of trials reporting CSS, DFS and PFS was limited, reducing the statistical power for these endpoints. Second, heterogeneity in surgical techniques, SLNB detection methods (e.g., dye vs. tracer combinations), and pathological ultrastaging protocols across studies may have introduced clinical variability, potentially affecting outcome comparability. Third, most included studies were retrospective in nature and subject to inherent selection bias. In particular, patients selected for SLNB may have had more favorable disease characteristics, which could confound the apparent non-inferiority of SLNB compared to LND. Furthermore, variations in follow-up duration and lack of uniform reporting on recurrence patterns limited the ability to assess long-term disease control comprehensively. Another limitation is that few studies stratified outcomes based on SLNB positivity status, limiting subgroup analysis for patients with micrometastasis or isolated tumor cells. The inclusion of single-arm studies, while valuable for evaluating long-term survival in SLNB-only cohorts, limits direct comparative interpretation.

Future prospective randomized controlled trials (RCTs) with standardized SLNB protocols and long-term follow-up are essential to validate the oncologic equivalence of SLNB. Studies should also evaluate cost-effectiveness, patient-reported outcomes, and the integration of SLNB with minimally invasive surgery or fertility-sparing approaches. Finally, the incorporation of molecular markers and artificial intelligence–assisted imaging may further enhance the accuracy and applicability of SLNB in cervical cancer.

## Conclusion

This meta-analysis demonstrates that SLNB is an oncologically safe and clinically viable alternative to systematic LND in early-stage cervical cancer. SLNB achieved comparable outcomes in CSS, DFS, OS, PFS, while significantly reducing postoperative complications. The inclusion of CSS as a primary endpoint and its confirmed robustness through sensitivity analysis further reinforces the reliability of SLNB in preserving cancer-related outcomes.

Although several studies have already contributed valuable evidence, further large-scale, well-controlled trials are needed to consolidate SLNB’s role across diverse clinical scenarios. These efforts will be essential to refine patient selection, standardize practice, and support broader implementation of SLNB as a standalone staging strategy in selected patients.

## Data Availability

The original contributions presented in the study are included in the article/supplementary material. Further inquiries can be directed to the corresponding author.
